# Trends and Outcomes of Gastrointestinal Bleeding Among Septic Shock Patients of the United States: A 10-Year Analysis of a Nationwide Inpatient Sample

**DOI:** 10.7759/cureus.8029

**Published:** 2020-05-08

**Authors:** Abdul Hasan Siddiqui, Moiz Ahmed, Tahir Muhammad Abdullah Khan, Saqib Abbasi, Saad Habib, Hafiz M Khan, Kartikeya Rajdev, Naureen Narula, Faraz Siddiqui

**Affiliations:** 1 Pulmonary and Critical Care Medicine, University of Illinois Urbana Champaign, Champaign, USA; 2 Gastroenterology, Icahn School of Medicine at Elmhurst Hospital Center, Elmhurst, USA; 3 Internal Medicine, Staten Island University Hospital/Northwell Health, Staten Island, USA; 4 Internal Medicine, Marshfield Medical Center, Marshfield, USA; 5 Hematology/Oncology, Staten Island University Hospital/Northwell Health, Staten Island, USA; 6 Gastroenterology and Hepatology, Guthrie Medical Group/Robert Packer Hospital, Sayre, USA; 7 Pulmonary and Critical Care Medicine, University of Nebraska Medical Center, Omaha, USA; 8 Pulmonary and Critical Care Medicine, Staten Island University Hospital/Northwell Health, Staten Island, USA; 9 Pulmonary and Critical Care Medicine, Robert Packer Hospital, Sayre, USA

**Keywords:** septic shock, gastrointestinal bleeding, coagulopathy, icu, mortality, nationwide inpatient sample, sepsis, hemorrhage

## Abstract

Introduction

Gastrointestinal bleeding (GIB) complicating septic shock (SS) presents a therapeutic challenge in intensive care units. Large-scale data regarding utilization, length of stay, and cost outcomes of this association are lacking.

Methods

We queried the Healthcare Cost and Utilization Project's Nationwide Inpatient Sample from 2003 to 2012, and identified all adult patients aged ≥18 years hospitalized for SS by the International Classification of Diseases, Ninth Revision (ICD-9) diagnostic code for SS and GIB. We compared the baseline characteristics and outcomes among patients with SS plus GIB to patients with SS without GIB.

Results

The weighted sample size from 2003 to 2012 was 119,684 admissions for SS. Among them, 6,571 (5.4%) patients were found to have a GIB. The mean age of the SS population with and without GIB was (mean/standard error of mean) [70.85 (0.43) vs. 67.43 (0.13) P < 0.001, respectively]. The incidence of GIB over the course of 10 years has remained stable; however, the mortality associated with GIB among SS patients is found to be declining especially from 2008 (59.2%) to 2012 (45.1%) (P < 0.01). Patients with SS and GIB compared to patients with SS and no GIB were found to have a longer length of stay [20.56 (0.61) vs. 15.76 (0.13) P < 0.001], higher mortality [54% vs. 45% P < 0.001], and higher admission costs in United States dollar ($) (mean/SEM) [$192,524.89 (7,378.20) vs. $142,688.55 (1,336.65) P < 0.001]. Univariate analysis demonstrated that comorbid conditions like peptic ulcer disease and cirrhosis had significant odds ratios {1.56 and 1.709, P = 0.016 and 0.046 respectively} for the occurrence of GIB with SS. Gastroesophageal reflux disease was found to be associated with a lower incidence of GIB [odds ratio: 0.57, P = 0.0008]. The cause of sepsis (pneumonia, urinary tract infection, or abdominal infections) was not a significant distinguishing factor for the incidence of GIB in SS.

Conclusion

GIB continues to affect the patients with SS admitted in intensive care units in the United States. We found an incidence of 5.4% of GIB in patients with SS, and it was associated with worse outcomes.

## Introduction

Gastrointestinal bleeding (GIB) is one of the major diagnoses of critical care patients. The incidence of GIB has been shown to be approximately 1.5% to 8.5% in critically ill patients, where patients with critical illness in conjunction with GIB exhibit higher mortality in comparison to those without GIB [1-4]. Over the last few decades, although the incidence of GIB in critically ill patients has been declining, the majority of thes data are obtained from postoperative critical care units. Thus, there is no clear consensus regarding the incidence and predictors of GIB in patients with septic shock (SS) [2,5,6]. Altemeier et al. were among the first to study the association between sepsis and the occurrence of GIB [7]. Of the 54 patients with combined GIB and sepsis, the majority of the patients exhibited gram-negative septicemia. In this study, while the authors anecdotally described a patient with SS, the overall number of patients experiencing SS was not explicitly mentioned. Nevertheless, stress-related mucosal damage (SRMD) was thought to be a possible etiology of GIB, while the mortality was much higher in this population (i.e., 69%) [7]. More recently, Cook et al. showed that although patients with sepsis and hypotension exhibited significantly higher odds of GIB using a simple variation analysis, these two conditions were not statistically significant when using a multivariate regression [3,8].

Mechanical ventilation and coagulopathy were identified as the major risk factors for GIB in critically ill patients, thereby prompting the Surviving Sepsis Campaign to recommend stress ulcer prophylaxis (SUP) in this group of patients (grade 1A). The recommendation for severe sepsis and SS is relatively weaker (grade 1B) owing to the low quality of evidence and lack of studies that analyze the association between sepsis and GIB, as previously described. The Surviving Sepsis Campaign recommends the use of proton pump inhibitors (PPI) over antihistamine 2 (H2) receptor blockers in high-risk patient populations (grade 2C) [8,9]. This was considered to be a weak recommendation and was challenged by multiple ensuing studies, which demonstrated that (1) there was no significant incidence of GIB in septic patients and (2) the use of prophylaxis with PPI resulted in an increased rate of GIB in comparison to H2 blockers [10,11]. Therefore, a clear idea of the incidence and outcomes of GIB in SS patients in a large sample would be detrimental to understanding the gravity of this association and could help to determine the need for SUP in these patients.

To the best of our knowledge, there is no large-scale data set available that analyzes the trends and outcomes of in-hospital GIB in patients with SS. Hence, by utilizing National Inpatient Sample (NIS) data, we aimed to (1) determine the incidence of GIB in patients who were admitted for SS and analyze trends in the United States over a period of 10 years, (2) determine the comorbidities that contribute to an increased risk of GIB, and (3) evaluate the length of hospital stay, cost, and mortality in SS patients with GIB.

## Materials and methods

Data source

We conducted a retrospective longitudinal study looking at all adult patients (>18 years) who were admitted in all US hospitals with a diagnosis of SS. The NIS database, developed by the Health Care Cost and Utilization Project (HCUP), was utilized to collect data from 2003 to 2012. The NIS is the largest publicly available, all-payer inpatient discharge database in the United States and represents stratified data from 20% of community hospitals in the United States. The NIS has been used in the past to study the trends and outcomes of various diseases and procedures [12-14]. It is composed of de-identified information on individual hospitalization, including demographics, insurance status, comorbidities, admission status, discharge diagnoses, procedures, outcomes, length of stay, and the cost of hospitalization. This de-identified data have been utilized in previous studies to study national trends for various in-hospital procedures and diagnoses, the cost of each hospitalization, the various outcomes, healthcare access, and disparities in care.

Study population

Using the International Classification of Diseases, Ninth Revision, Clinical Modification (ICD-9-CM) codes, we queried the Healthcare Cost and Utilization Project’s Nationwide Inpatient Sample from 2003 to 2012. We identified all adult patients aged >18 years who had been hospitalized for SS according to the following criteria (which were previously validated by a recent study that used data from the same database [BMC Infectious diseases 2016 10.1186]): (1) primary ICD-9 diagnosis of infection associated with sepsis plus vasopressor use, (2) primary ICD-9 diagnosis of infection associated with sepsis plus a non-primary diagnosis of SS, or (3) primary ICD-9 diagnosis of SS [15]. Hospitalizations with missing data for age, gender, admission, or discharge diagnosis, or mortality were excluded. We, therefore, analyzed the most common etiologies of SS, which included pneumonia, urinary tract infections, and abdominal infections.

Definition of variables

The NIS variables were utilized to identify patient age, gender, and race. Age was divided into five categories, <20 years, 20-39 years, 40-59 years, 60-79 years, and >80 years. Race was further divided into white, black, Hispanic, Asian, Pacific Islander, or Native Americans. A modified Charlson Comorbidity Index (CCI) was used to define the severity of the comorbid conditions. The scores range from 0 to 33, where a higher score corresponds to a greater burden of comorbid disease. The cost of each hospitalization was calculated by merging the data with the cost to charge ratio from the HCUP database, and this was standardized and adjusted for each year. The cost of each year was adjusted in reference to the 2016 US dollar value by using the consumer price index to account for inflation.

Outcomes

The following outcomes were analyzed in the present study. (1) Our primary objective was to examine the incidence of GIB in patients who were hospitalized with SS in the United States between 2003 and 2012. (2) Our secondary objective was to investigate the independent predictors of GIB in patients with SS. Some of the well-established comorbidities associated with GIB, such as alcoholism, peptic ulcer disease (PUD), underlying liver disease, atrial fibrillation (AF), congestive heart failure, valvular heart disease, renal failure, anemia, previous significant bleeding, coagulation disorders, and gastrointestinal malignancies (i.e., esophageal, gastric, small intestinal, large intestinal, and rectosigmoid cancers) were also included in our analysis. Established risk factors for the development of SS (e.g., pneumonia, urinary tract infections, and abdominal infections) were also studied. (3) We compared the length of stay (LOS) and cost per hospitalization for patients with SS and GIB versus patients with SS without GIB. (4) Mortality rates between the two groups were also assessed. 

Statistical analyses

Using the NIS database, SS was defined using the following ICD-9 diagnostic codes: (1) primary ICD-9 diagnosis of infection associated with sepsis plus vasopressor use, (2) primary ICD-9 diagnosis of infection associated with sepsis plus a non-primary diagnosis of SS, or (3) primary ICD-9 diagnosis of SS. GIB was defined using the corresponding clinical classification software codes: (1) a chi-square or ANOVA was used accordingly to identify the demographic differences between SS patients with and without GIB, (2) a two-sample t-test was used to assess the impact of GIB on in-hospital mortality, LOS, and total charges for SS patients with or without GIB, (3) univariate logistic regression analysis was used to calculate the odds ratios (OR) for causes of SS, common comorbid conditions, and their associations with the incidence of GIB, and (4) a multivariate analysis of significant comorbidities was then performed to account for any confounders among the significant comorbidities.

## Results

From 2002 to 2012, a total of 119,684 patients aged >18 years were hospitalized with the primary discharge diagnosis of SS. Out of 119,684 patients with SS, 6,571 (5.4%) suffered from GIB, and in-hospital mortality for these patients was significantly higher in comparison to patients without GIB (54 vs. 45%, respectively; P < 0.001).

**Figure 1:**
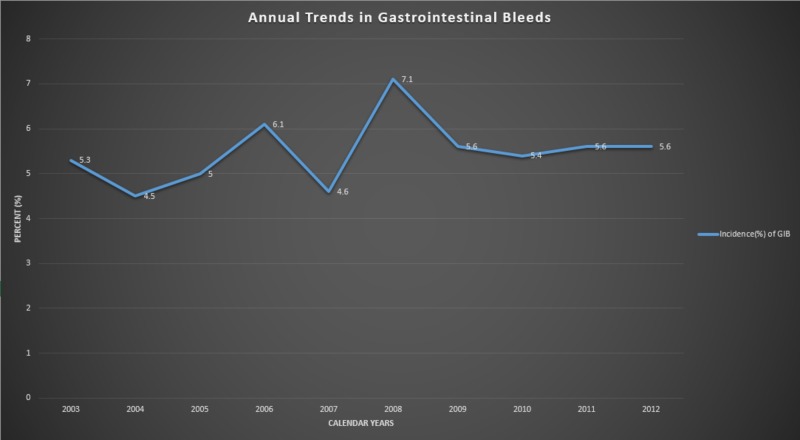
Annual trends in GIB in the United States from 2003 to 2012 appears to have gradually increased over the course of 10 years. GIB, gastrointestinal bleeding.

**Figure 2:**
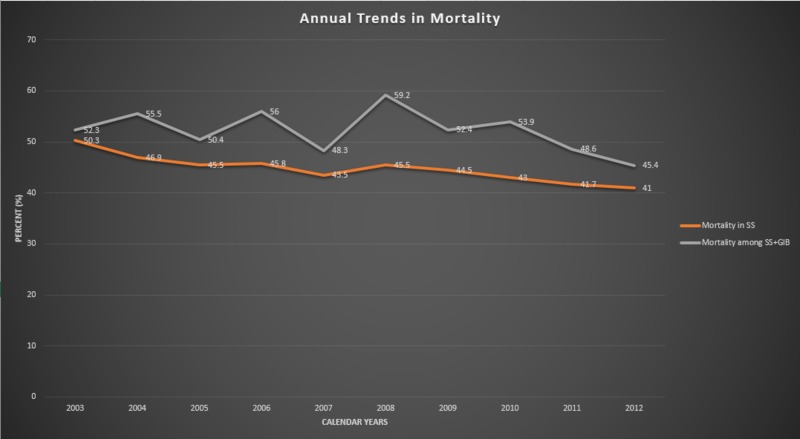
Comparison of annual mortality trends between SS alone and SS with GIB. Graph demonstrating that annual mortality is higher among patients with SS and GIB, but is trending down over the course of years. GIB, gastrointestinal bleeding; SS, septic shock.

Over the course of 10 years, the mean annual incidence of GIB among SS patients in the United States ranged from 4.5% to 7.1%, with the highest rate in 2008. However, since 2008, the incidence appears to have decreased and was stable until 2012. The overall prevalence was found to be 5.4%. Similarly, the mortality among these patients seems to have decreased since 2008, with the lowest death rate in 2012 (45%). Interestingly, not only was the incidence of GIB among SS patients highest in 2008, but it was also associated with the highest mortality rate in that year, and mortality was significantly increased in comparison to the SS population as a whole (59 vs. 46%, respectively; P < 0.05). The mortality rates of SS patients were found to continually decline over the course of ten years, from 50% in 2003 to the lowest rate in 2012 (41%) (Figures 1, 2).

Table 1 shows the baseline characteristics and demographic variables of patients admitted with SS, with and without in-hospital GIB. The mean age of patients with SS who suffered from GIB was 70.85 years (standard error of mean = 0.43), and the incidence of GI bleeds in these patients increased with increasing age. There were no significant gender differences noted in SS patients with and without GIB. Native American and black patients exhibited significantly higher adjusted rates of GIB in comparison to other races/ethnicities (6.9% and 6.88%, respectively; P < 0.001). White Americans had a 5.35% chance of suffering from GIB during SS.

Patients with SS complicated by GIB exhibited a significantly longer length of hospital stay of 20.56 days (standard deviation [SD] = 0.61; P < 0.001) in comparison to 15.76 days for patients with SS without GIB (SD = 0.13; P < 0.01). The mean hospital cost was also inflated in patients with GIB in comparison to patients without GIB ($192,524.89 vs. $142,688.55, respectively; P < 0.001). In-hospital mortality was significantly higher (54%) in SS patients with GIB in comparison to that of non-bleeders (45%).

**Table 1:** Baseline characteristics and demographics of septic shock patients with and without GI bleed. *P-value is significant for the age-related difference in the incidence of GI bleed. ** P-value is significant for the race-related difference in the incidence of GI bleed. GI, gastrointestinal; SE, standard error

Demographics		GI Bleed during hospitalization				P-value
		No		Yes		
		#	%	#	%	
Age group		586	98.65	8	1.35	<0.001 *
	<20					
	20–39	1,026	97.53	26	2.47	
	40–59	4,905	95.02	257	4.98	
	60–79	10,127	94.33	609	5.67	
	≥80	6,316	93.61	431	6.39	
Age (mean/SE)		67.43 (0.13)		70.85 (0.43)		<0.001
Race (uniform)		13,877	94.65	785	5.35	<0.001**
	White					
	Black	2,178	93.12	161	6.88	
	Hispanic	1,901	94.02	121	5.98	
	Asian or Pacific Islander	569	94.05	36	5.95	
	Native American	135	93.1	10	6.9	
	Other	508	92.53	41	7.47	
Gender		9,781	94.4	580	5.6	0.6537
	Female					
	Male	9,387	94.24	574	5.7	
Length of stay (mean/SE)		15.76 (0.13)		20.56 (0.61)		<0.001
In-hospital death (mean/SE)		0.45 (0.00)		0.54 (0.01)		<0.001
Total charges (mean/SE)		142,688.55 (1336.65)		192,524.89 (7378.20)		<0.001

Table 2 shows the various comorbidities and their associated risk of GIB in the form of ORs. Patients with pre-existing gastrointestinal conditions such as PUD and cirrhosis had a higher propensity of developing GIB with a likelihood ratio of 1.7 (95% Wald confidence interval [CI] 1.003-2.914) and 1.55% (95% CI 1.12-2.16), respectively. However, patients with a history of gastroesophageal reflux disease (GERD) exhibited a lower likelihood of developing GIB during SS, 0.567 (95% Wald CI 0.407-0.790). Even though end-stage renal disease patients were found to have a higher likelihood of developing GIB, the results were not statistically significant, 1.247 (95% Wald CI 0.988-1.573; P = 0.0628). On the other hand, SS patients with comorbidities such as AF, valvular heart disease, coronary artery disease, hypertension, chronic obstructive pulmonary disease, rheumatoid arthritis, diabetes mellitus, and tobacco use had relatively low odds of having GIB. The multivariate analysis also showed that PUD, cirrhosis, mechanical ventilation, and coagulopathy conferred a significantly higher chance of GIB in SS patients. The CCI > 2 was not significantly associated with GIB (OR: 0.857; 95% Wald CI 0.648-1.133; P = 0.278).

**Table 2:** Risk factors associated with gastrointestinal bleeding in patients with septic shock. CCI, Charlson Comorbidity Index

Comorbidity	Odds ratio	95% Wald confidence limits		P-value
Gastroesophageal reflux disease	0.567	0.407	0.79	0.0008
Atrial fibrillation	1.006	0.876	1.154	0.9344
Cirrhosis	1.556	1.121	2.161	0.0127
Peptic ulcer disease	1.709	1.003	2.914	0.0462
Rheumatoid arthritis	0.8	0.483	1.324	0.3834
Valvular heart disease	0.848	0.645	1.116	0.238
Coronary artery disease	0.722	0.603	0.864	0.0004
Hypertension	0.836	0.74	0.944	0.0039
Chronic obstructive pulmonary disorder	1.009	0.887	1.148	0.8921
Diabetes	0.78	0.668	0.912	0.0018
Tobacco use disorder	0.472	0.325	0.685	<0.0001
Pancytopenia	1.079	0.734	1.587	0.6987
End-stage renal disease	1.247	0.988	1.573	0.0628
Mechanical ventilation	1.451	1.228	1.635	<0.001
Congestive heart failure	1.006	0.89	1.138	0.9194
Anemia	1.166	1.022	1.331	0.0226
Peripheral vascular disease	0.872	0.663	1.148	0.3716
Transient ischemic attack/stroke	1.459	1.165	1.827	0.001
Weekend admission	1.033	0.91	1.172	0.6179
Coagulopathy	1.342	1.166	1.544	<0.0001
Esophageal cancer	0.584	0.142	2.392	0.4545
Stomach cancer	1.176	0.365	3.792	0.7862
Colon cancer	1.192	0.755	1.882	0.4509
CCI ≥2	0.857	0.648	1.133	0.2786

Table 3 shows the common etiologies of SS and their impact on the incidence of GIB. The etiology of the SS itself did not have an independent effect on the occurrence of GIB, as the ORs were similar for pneumonia, abdominal infections, and urinary tract infections.

**Table 3:** Common etiologies of septic shock and their risk for gastrointestinal bleeding.

Cause	Odds ratio	95% Wald confidence limits		P-value
Pneumonia	1.072	0.955	1.204	0.2361
Urinary tract infection	0.96	0.783	1.176	0.6905
Abdominal infection	0.921	0.808	1.05	0.219

## Discussion

In the present study, the overall incidence of GIB in SS patients was 5.4%. This is relatively higher in comparison to incidences reported for critically ill patients in some recent studies, where clinically important cases of GIB occurred in 2.6% of the patients (95% Wald CI 1.6­%-3.6%) [6]. A study conducted by Pimentel et al. showed that in the era of increasing stress ulcer prophylaxis, the incidence could be as low as 0.17% in critically ill patients. However, these were all endoscopically proven cases of GIB, while some patients with GIB included in the present study may not have undergone an endoscopic procedure during index admission [16]. Nevertheless, in agreement with previous studies, our data correlate with the incidence of GIB in patients with a critical illness. Furthermore, Skillman et al. reported a 5% incidence of fatal stress ulceration in 150 patients who were admitted to the ICU. Sepsis, respiratory failure, and hypotension were the common risk factors identified for GIB in critical care patients [17,18].

The higher incidence of GIB observed in cases of SS could be partially a result of the severity of SS, which causes hypoperfusion, or perhaps the use of vasopressors in SS patients, which causes splanchnic ischemia and leads to SRMD [19]. Furthermore, SS is one of the most common admitting diagnoses in medical ICUs, whereas the majority of previous studies on GIB were conducted using postoperative critical care units.

A decline in the incidence of GIB in critical care patients has been reported in previous studies and is attributed to the utilization of aggressive prophylactic measures in ICU used in US hospitals [16,20]. A detailed analysis of the existing literature reflects improvements in the guidelines for inpatient care in ICU settings for stress ulcer prophylaxis, which includes early aggressive resuscitation. However, the mortality associated with GIB in SS patients is declining. Moreover, this decline in mortality is more obvious over the latter period of the study, especially from 2009 to 2012. We could not establish an exact cause for this observation owing to the inherent limitations of the NIS database.

In agreement with an earlier study by Cook et al., in-hospital mortality in SS patients with GIB was significantly higher than in patients without GIB (54 vs. 45%; P < 0.001) [3]. Although Krag et al. demonstrated that clinically important GIB is not associated with increased adjusted 90-day mortality, this finding was explained by the relatively increased severity of comorbid illnesses (e.g., multiple organ failures and increased age in patients with GIB). Subsequently, Cook et al. observed that upper GIB exerted an independent adverse impact on both morbidity and mortality (i.e., the relative risk of death of up to 4% in critically ill patients), and also resulted in an excessive LOS in ICUs of four to eight days [3]. These findings are in agreement with the results for SS patients in our study.

In addition, we found that cirrhosis, PUD, coagulopathy, and mechanical ventilation were associated with significantly higher ORs of GIB, and therefore could be considered as risk factors for GIB in SS patients. This is similar to risk factors identified in previous studies on critical care patients [2,20-23]. Interestingly, the CCI was not associated with GIB in SS patients in our study, which showed that a SS patient with a CCI > 2 was less likely to have GIB. These results are in contrast to other aforementioned studies and could be attributed to the fact that SS patients with a CCI > 2 have the highest mortality rates, and that patients who fall into this category may die from the severity of their underlying illnesses before developing GIB.

Gastroesophageal reflux, nasogastric (NG) tubes, and duodenogastroesophageal (bile) reflux are reported to be potential mechanisms of esophageal injury in patients with critical illness [24]. On the contrary, our study suggests that a history of GERD does not predispose patients with SS to a higher OR of GIB (0.567; P = 0.0008). This finding is in agreement with that of Wilmer et al., who studied mechanically ventilated patients and recognized chemical insult by bile reflux and mechanical irritation by direct NG tube trauma as the major factors involved in the pathogenesis of esophageal mucosal injury [25]. It could be presumed that while patients with a history of GERD were already using anti-reflux therapies, which could have offered protective benefits, we were not able to derive the information from the NIS database to support this assumption.

Although previous studies have hinted that gram-negative sepsis and peritonitis were more commonly associated with GIB in SS patients, we were not able to ascertain the exact microbiological etiology of SS and its relationship to GIB. However, we found no significant differences in the occurrence of GIB in relation to the specific source of sepsis, be it pneumonia, urinary tract infection, or abdominal infections. This suggests that the intense inflammatory reaction that is characteristic of SS is more likely to be related to the occurrence of GIB than the source of infection. This was consistent with the findings of Krag et al., who showed that both extra-abdominal sepsis and peritonitis were independent risk factors for GIB in critically ill patients [2].

We do acknowledge that, similarly to other NIS studies, the present study has major limitations. First, although ICD-9-CM codes for GIB and SS have been previously validated and used, we cannot exclude the possibility of variations in the accuracy of coding between hospitals. While we did try to include all available codes for SS in the ICD-9 code classification in order to include all possible subgroups of SS patients, the same cannot be said about the GIB patients. Secondly, our inability to ascertain the use of GI prophylaxis, steroid use, vasopressor use, the amount of blood transfusion per patient, and endoscopic intervention in the included patients, which could have potentially confounded the results in relation to the outcomes, was limited in the present study. Lastly, long-term outcomes could not be evaluated. Therefore, as a result of the inherent limitations associated with retrospective observational studies, although associations between risk factors can be obtained, no inferences can be made regarding causation.

Despite these limitations, we believe that our study, which used a large sample size, provides critical information regarding the long-term trends and outcomes related to GIB in SS patients. We can clearly see that, unlike other critical care patients, a significant number of SS patients are affected by a significant occurrence of GIB, and even though the mortality related to GIB appears to be decreasing, these patients still carry a high risk of morbidity and mortality compared to those without GIB. Although the recent data that endorse stress ulcer prophylaxis in severe sepsis/SS are controversial, our study warrants further large-scale prospective studies in order to elucidate whether SS is an independent risk factor of GIB, as well as the type and duration of stress ulcer prophylaxes needed to prevent stress ulcer bleeding in SS patients [26].

## Conclusions

Among the population of the United States admitted between 2003 and 2012, GIB among SS patients appears to be a significant concern. In our study, an incidence of GIB of 5.4% was observed in patients with SS and was significantly associated with increased mortality, LOS, and cost burden among SS patients. The incidence of GIB among SS appears to be gradually increasing, but the annual mortality associated with GIB among SS appears to be declining over the course of years. It will be interesting to observe this trend over the next decade.

Patients with PUD and cirrhosis are at the highest risk of developing GIB, and appropriate prophylaxes for these patients could prevent the morbidity and mortality associated with GIB in SS patients. Further randomized controlled trials are warranted to study the effects of SUP among high-risk population with SS.
